# Transparency of Values in Science: Elliott and Resnik Respond

**DOI:** 10.1289/ehp.1408936R

**Published:** 2014-11-01

**Authors:** Kevin C. Elliott, David B. Resnik

**Affiliations:** 1 Michigan State University, East Lansing, Michigan, USA; 2 National Institute of Environmental Health Sciences, National Institutes of Health, Department of Health and Human Services, Research Triangle Park, North Carolina, USA

We thank Schwartz for his thoughtful comments, which provide an opportunity for us to clarify some of the points in our commentary, in which we called for greater transparency about the values that influence policy-relevant research (Elliott and Resnik 2014). His first concern is that even though we call for employing different standards of evidence in various social contexts, the evidence itself remains the same. We agree; in our commentary we were not claiming that the evidence itself changes but only that the form of evidence required for different decisions depends on the social context. Nevertheless, it is important to recognize that although the evidence itself does not change, it has to be interpreted and weighed, and many contemporary science policy disputes stem from disagreements about how to do so (Douglas 2012). Therefore, our view is that conflicts over public policies often stem from value judgments about both the nature of the evidence and standards of evidence. We agree with Schwartz about the solution: Transparency is essential. The more scientists acknowledge the assumptions and values that influence their interpretations of evidence and their decisions about how to weigh it, the better.

Schwartz’s second point is that it is problematic for us to treat tort law and chemical regulations as equivalent contexts, in which weaker standards of evidence can appropriately inform policy. We agree that different standards of evidence may be appropriate in the two contexts. However, we caution against equating the standards of evidence expected in tort law with those expected in more traditional scientific contexts. The tort system requires only a preponderance of evidence (> 50% likelihood) to win a case; this is much weaker evidence than scientists typically demand when presenting or publishing results, and confusion about these differing standards has led to significant legal controversies (Cranor 2006).

Schwartz’s third point is that other conflicts of interest, such as “ideological ties to advocacy organizations,” are important to disclose in addition to financial ties to industry. We heartily agree; indeed, in our commentary (Elliott and Resnik 2014) we stated that “Disclosures of competing financial interests and nonfinancial interests (such as professional or political allegiances) also provide opportunities for more transparent discussions” (Elliott and Resnik 2014). One of the aims of our commentary was to encourage more careful thinking about how to promote transparency regarding a wide range of different factors that could influence scientists’ reasoning, including ideology. Nevertheless, there are at least two reasons that financial connections to industry groups should continue to receive careful attention: *a*) Advocacy organizations typically have much fewer resources than industry to generate policy-relevant research that serves their interests (Elliott 2011); and *b*) a large body of evidence indicates that industry funding has important effects on research outcomes, whereas there is less information about how ideological ties affect research (e.g., Lundh et al. 2012).

Schwartz’s fourth concern is that it is important to think about risk–benefit relationships when applying science to public policy. This is a very good point, but it needs to be considered in conjunction with the fact that scientists’ values and assumptions may influence their assessments of the evidence. It would be ideal if scientists could provide perfectly unbiased risk assessments to policy makers, who could then evaluate the risks versus the benefits in order to make policy decisions. Unfortunately, this picture is unrealistic; scientists’ views about the benefits associated with new technologies likely have implicit influences on their risk assessments. For example, evidence from the literature on risk perception indicates that people’s perceptions of risk are influenced by a number of factors, including the voluntariness of the risks, the fairness of their distribution, their familiarity, and the perceived benefits associated with them (Fischhoff et al. 1981). Scientists are subject to these same influences, especially when they have limited data or are forced to weigh multiple forms of evidence (Cooke 1991). This provides further support for our central claim, namely, that scientists should explore ways to acknowledge the values that may influence them rather than denying the presence of these influences.
